# Supplementary material for: Method Validation for Determination of Thallium by Inductively Coupled Plasma Mass Spectrometry and Monitoring of Various Foods in South Korea
Source: Molecules. 2021 Nov 6;26(21):6729. doi: 10.3390/molecules26216729 (PMC8588170; doi:10.3390/molecules26216729)

Table S1. Evaluation of intraday and interday accuracy and precision at low, medium, and high concentrations of thallium (Tl) in representative foods.

	**Intra-day**	**Day 1 (n = 7)**		**Day 2 (n = 7)**		**Day 3 (n = 7)**		**Inter-day (n = 3)**	
**Representative food**	**Nominal Concentration (μg kg** $^{-1}$**)**	Accuracy (%) [a]	Precision (%RSD) [b]	Accuracy (%) [a]	Precision (%RSD) [b]	Accuracy (%) [a]	Precision (%RSD)	Accuracy (%) [a]	Precision (%RSD) [b]
Rice	0.1	119.81	3.97	119.54	1.98	112.11	3.16	110.44	1.20
	0.5	102.15	2.66	104.47	2.22	103.89	2.28	100.34	5.29
	1.0	107.77	1.58	113.71	5.74	108.69	1.32	102.98	4.52
Apple	0.1	102.22	4.46	95.00	5.19	105.04	5.68	103.04	2.19
	0.5	97.99	0.99	101.63	1.38	102.70	2.02	98.33	1.56
	1.0	112.55	4.25	95.45	3.75	96.65	2.04	94.75	1.31
Beef	0.1	109.86	3.09	107.11	3.17	108.17	2.56	109.99	2.41
	0.5	91.10	1.79	91.12	2.79	99.36	1.59	98.71	4.57
	1.0	82.06	1.63	83.96	0.88	99.71	2.83	97.13	6.99
Sea salt	0.1	82.39	4.43	83.21	3.36	83.77	5.39	94.94	9.79
	0.5	87.22	4.85	87.71	2.06	94.84	6.85	92.05	1.09
	1.0	90.86	6.28	97.08	3.32	106.23	9.08	101.42	9.49
Orange juice	0.1	114.12	2.96	104.86	1.58	112.61	5.22	107.30	7.45
	0.5	89.19	1.98	105.24	3.02	100.70	2.03	102.71	5.99
	1.0	113.07	1.70	100.00	1.52	94.94	2.05	96.11	6.20
Sesame oil	0.1	116.62	2.44	110.30	5.09	117.98	3.80	107.54	6.12
	0.5	114.11	3.44	114.77	3.10	118.89	2.48	107.48	5.61
	1.0	115.62	2.16	120.00	1.31	119.95	2.30	103.70	4.61

[a] (Mean measured concentration / nominal concentration) × 100; [b] (Standard deviation / mean) × 100); RSD = relative standard deviation.

Table S2. Tl content (fresh weight) in specific items of agricultural products.

Category (n)	Item (n)	Min – Max (μg kg $^{-1}$)	Mean ± SD (μg kg $^{-1}$)
Grains (32)	Rice (3)	0.00 – 0.00	0.00 ± 0.00
	Brown rice (3)	0.43 – 0.47	0.45 ± 0.01
	Barley (3)	0.00 – 0.00	0.00 ± 0.00
	Corn (4)	0.00 – 0.53	0.14 ± 0.23
	Glutinous millet (3)	3.46 – 4.39	4.07 ± 0.43
	Sorghum (3)	0.66 – 5.27	3.02 ± 1.88
	Oat (4)	0.00 – 0.00	0.00 ± 0.00
	Glutinous rice (3)	0.00 – 0.45	0.15 ± 0.21
	Proso millet (3)	0.00 – 0.49	0.30 ± 0.21
	Adlay (3)	0.44 – 0.83	0.62 ± 0.16
Beans (10)	Soybean (3)	0.00 – 2.31	1.32 ± 0.97
	Black bean (3)	0.00 – 0.89	0.30 ± 0.42
	Kidney bean (4)	0.00 – 0.00	0.00 ± 0.00
Fruits (9)	Apple (3)	0.02 – 0.47	0.28 ± 0.19
	Pear (3)	0.83 – 7.00	4.14 ± 2.54
	Mandarin (3)	0.02 – 0.02	0.02 ± 0.00
Tubers (6)	Potato (3)	3.78 – 4.34	4.04 ± 0.23
	Sweet potato (3)	5.92 – 13.21	9.29 ± 3.00
Mushrooms (6)	King oyster mushroom (3)	0.02 – 0.02	0.02 ± 0.00
	Winter mushroom (3)	0.02 – 0.02	0.02 ± 0.00
Root vegetables (19)	White radish (3)	2.74 – 7.11	5.08 ± 1.80
	Carrot (3)	0.02 – 2.06	0.86 ± 0.87
	Garlic (4)	0.02 – 3.28	1.28 ± 1.22
	Onion (3)	0.02 – 0.40	0.26 ± 0.17
	Burdock root (3)	0.02 – 0.78	0.28 ± 0.35
	Ginseng (3)	1.46 – 6.85	4.32 ± 2.22
Leafy vegetables (19)	Perilla leaf (3)	0.02 – 0.34	0.23 ± 0.14
	Chinese cabbage (3)	0.20 – 0.47	0.34 ± 0.11
	Lettuce (3)	0.11 – 13.63	4.69 ± 6.32
	Spinach (3)	0.80 – 4.53	2.26 ± 1.63
	Cabbage (4)	0.02 – 15.93	4.13 ± 6.82
	Spring onion (3)	0.17 – 0.93	0.55 ± 0.31
Other vegetables (12)	Eggplant (3)	0.02 – 0.49	0.22 ± 0.27
	Red pepper (3)	0.02 – 0.60	0.02 ± 0.00
	Zucchini (3)	0.02 – 0.02	0.02 ± 0.00
	Cucumber (3)	0.02 – 0.02	0.02 ± 0.00

[a] n is number of samples; [b] results < MLOD were set equal to 1/2 MLOD values.

Table S3. Tl content (fresh weight) of specific items in livestock products.

Category (n)[a]	Item (n)[a]	Min – Max (μg kg $^{-1}$)	Mean ± SD (μg kg $^{-1}$)
Eggs (3)	Eggs (3)	0.65 – 5.72	2.46 ± 2.31
Meats (9)	Chicken (3)	0.62 – 1.63	1.19 ± 0.42
	Pork (3)	0.54 – 0.65	0.59 ± 0.05
	Beef (3)	0.51 – 0.67	0.58 ± 0.07

[a] n is number of samples; [b] results < MLOD were set equal to 1/2 MLOD values.

Table S4. Tl content (fresh weight) of specific items in fishery products.

Category (n)[a]	Item (n)[a]	Min – Max[b] (μg kg $^{-1}$)	Mean ± SD[b] (μg kg $^{-1}$)
Fishes (21)	Hairtail (3)	0.81 – 0.97	0.90 ± 0.07
	Mackerel (3)	1.04 – 1.18	1.12 ± 0.06
	Flatfish (3)	0.41 – 0.89	0.62 ± 0.20
	Cod (3)	0.57 – 1.22	0.96 ± 0.28
	Walleye pollock (3)	1.03 – 1.21	1.12 ± 0.07
	Spanish mackerel (3)	0.76 – 1.79	1.44 ± 0.48
	Small yellow croaker (3)	0.91 – 1.87	1.24 ± 0.45
Cephalopoda (6)	Squid (3)	1.07 – 1.10	1.08 ± 0.02
	Octopus (3)	1.04 – 1.22	1.11 ± 0.08
Shellfishes (10)	Oyster (3)	1.20 – 2.05	1.65 ± 0.35
	Manila clam (4)	1.30 – 6.02	3.32 ± 1.94
	Mussel (3)	1.70 – 3.17	2.43 ± 0.60
Crustaceans (4)	Crab (4)	1.07 – 1.79	1.28 ± 0.29
Sea algae (9)	Laver (3)	0.02 – 5.21	1.75 ± 2.44
	Sea mustard (3)	0.02 – 12.72	4.26 ± 5.99
	Hijiki (3)	0.02 – 0.02	0.02 ± 0.00

[a] n is number of samples; [b] results < MLOD were set equal to 1/2 MLOD values.

Table S5. Tl content (fresh weight) of specific items in beverages.

**Category (n)**[a]	**Item (n)**[a]	**Min – Max**[b] **(μg kg** $^{-1}$**)**	**Mean ± SD**[b] **(μg kg** $^{-1}$**)**
Infusion teas (18)	Mate tea (3)	0.77 – 1.99	1.50 ± 0.53
	Solomon-seal root tea (3)	0.72 – 1.61	1.17 ± 0.36
	Black tea (3)	0.47 – 0.73	0.58 ± 0.11
	Barley tea (3)	0.01 – 0.50	0.17 ± 0.23
	Brown rice tea (3)	0.01 – 0.60	0.21 ± 0.28
	Buckwheat tea (3)	0.01 – 0.01	0.01 ± 0.00
Other beverages (21)	Red ginseng drink (3)	1.30 – 4.12	2.49 ± 1.19
	Fruit juice (3)	0.60 – 0.73	0.68 ± 0.06
	Soybean milk (3)	0.01 – 0.70	0.24 ± 0.33
	Corn silk drink (3)	0.01 – 0.01	0.01 ± 0.00
	Sports drink (3)	0.01 – 0.01	0.01 ± 0.00
	Instant coffee (3)	0.01 – 0.01	0.01 ± 0.00

[a] n is number of samples; [b] results < MLOD were set equal to 1/2 MLOD values.

Table S6. Tl content (fresh weight) of specific items in other processed foods.

Category (n)[a]	Item (n)[a]	Min – Max[b] (μg kg $^{-1}$)	Mean ± SD[b] (μg kg $^{-1}$)
Snacks (6)	Potato crisp (3)	1.59 – 6.73	3.49 ± 2.30
	Fruit jelly (3)	0.45 – 0.60	0.51 ± 0.07
Salted processed seafood (12)	Fish cake (3)	0.94 – 1.41	1.19 ± 0.19
	Fermented pollock roe (3)	0.01 – 0.01	0.01 ± 0.00
	Fermented squid (3)	0.01 – 0.01	0.01 ± 0.00
	Seasoned laver (3)	0.01 – 0.01	0.01 ± 0.00
Pickled foods (12)	Radish kimchi (3)	0.01 – 19.46	1.63 ± 5.38
	Chinese cabbage kimchi (3)	0.01 – 0.01	0.01 ± 0.00
	Spring onion kimchi (3)	0.01 – 0.01	0.01 ± 0.00
	Pickled radish (3)	0.01 – 0.01	0.01 ± 0.00
Sauces (18)	Soybean sauce (3)	0.01 – 0.01	0.01 ± 0.00
	Red pepper paste (3)	0.01 – 0.01	0.01 ± 0.00
	Fermented soybean paste (3)	0.01 – 0.01	0.01 ± 0.00
	Extra-fermented soybean paste (3)	0.01 – 0.01	0.01 ± 0.00
	Black soybean paste (3)	0.01 – 0.01	0.01 ± 0.00
	Mixed seasoning paste (3)	0.01 – 0.01	0.01 ± 0.00
Oil products (27)	Corn oil (3)	0.01 – 0.01	0.01 ± 0.00
	Olive oil (3)	0.01 – 0.01	0.01 ± 0.00
	Rape seed oil (3)	0.01 – 0.01	0.01 ± 0.00
	Sesame oil (3)	0.01 – 0.01	0.01 ± 0.00
	Soybean oil (3)	0.01 – 0.01	0.01 ± 0.00
	Sunflower seed oil (3)	0.01 – 0.01	0.01 ± 0.00
	Grape seed oil (3)	0.01 – 0.01	0.01 ± 0.00
	Perilla oil (3)	0.01 – 0.01	0.01 ± 0.00
	Magarine (3)	0.01 – 0.01	0.01 ± 0.00
Dairy products (6)	Butter (3)	0.01 – 0.01	0.01 ± 0.00
	Yogurt (3)	0.01 – 0.01	0.01 ± 0.00
Grain products (3)	Flour (3)	0.00 – 0.00	0.00 ± 0.00
Livestock processed foods (3)	Ham (3)	0.01 – 0.01	0.01 ± 0.00
Noodles (3)	Instant noodles (Ramen) (3)	0.01 – 0.01	0.01 ± 0.00

[a] n is number of samples; [b] results < MLOD were set equal to 1/2 MLOD values.

Table S7. Estimated dietary exposure to Tl by age (Lower Bound).

Food Group		Exposure (μg kg $^{-1}$ kw day^{-1})					
		**0 – 2 years**	**3 – 6 years**	**7 – 12 years**	**13 – 19 years**	**20 – 64 years**	**65 years and over**
Agricultural products	Cereals	0.0000-0.0002	0.0000-0.0001	0.0000-0.0001	0.0000-0.0001	0.0000-0.0001	0.0000-0.0001
	Fruits	0.0000-0.0064	0.0000-0.0036	0.0000-0.0014	0.0000-0.0008	0.0000-0.0010	0.0000-0.0013
	Beans	0.0000-0.0001	0.0000-0.0001	0.0000-0.0001	0.0000-0.0001	0.0000-0.0001	0.0000-0.0002
	Mushrooms	0.0000	0.0000	0.0000	0.0000	0.0000	0.0000
	Tubers	0.0035-0.0119	0.0021-0.0047	0.0012-0.0027	0.0006-0.0018	0.0011-0.0026	0.0009-0.0043
	Vegetables	0.0000-0.0055	0.0000-0.0031	0.0000-0.0028	0.0000-0.0023	0.0000-0.0031	0.0000-0.0032
Livestock products	Eggs	0.0012-0.0104	0.0010-0.0086	0.0006-0.0052	0.0003-0.0028	0.0003-0.0027	0.0002-0.0016
	Meats	0.0001-0.0013	0.0002-0.0012	0.0002-0.0016	0.0001-0.0015	0.0001-0.0008	0.0000-0.0002
Fishery products	Crustaceans	0.0000-0.0000	0.0000-0.0000	0.0000-0.0000	0.0000-0.0000	0.0000-0.0000	0.0000-0.0000
	Fishes	0.0000-0.0002	0.0000-0.0001	0.0000-0.0001	0.0000-0.0000	0.0000-0.0001	0.0000-0.0001
	Cephalopoda	0.0000-0.0001	0.0000-0.0001	0.0000-0.0001	0.0000-0.0001	0.0000-0.0001	0.0000-0.0001
	Shellfishes	0.0000-0.0004	0.0000-0.0003	0.0000-0.0001	0.0000-0.0001	0.0000-0.0001	0.0000-0.0001
	Sea algae	0.0000-0.0006	0.0000-0.0004	0.0000-0.0002	0.0000-0.0001	0.0000-0.0002	0.0000-0.0002
Processed products	Beverages	0.0000-0.0017	0.0000-0.0016	0.0000-0.0007	0.0000-0.0006	0.0000-0.0003	0.0000-0.0001
	Other processes products	0.0000-0.0003	0.0000-0.0005	0.0000-0.0009	0.0000-0.0005	0.0000-0.0014	0.0000-0.0016

Table S8. Estimated dietary exposure to Tl by age (Upper Bound).

Food Group		Exposure (μg kg $^{-1}$ kw day $^{-1}$)					
		0 – 2 Years	3 – 6 Years	7 – 12 Years	13 – 19 Years	20 – 64 Years	Over 65 Years
Agricultural products	Cereals	0.0000-0.0002	0.0000-0.0001	0.0000-0.0001	0.0000-0.0001	0.0000-0.0001	0.0000-0.0001
	Fruits	0.0000-0.0064	0.0000-0.0036	0.0000-0.0014	0.0000-0.0008	0.0000-0.0010	0.0000-0.0013
	Beans	0.0000-0.0001	0.0000-0.0001	0.0000-0.0001	0.0000-0.0001	0.0000-0.0001	0.0000-0.0002
	Mushrooms	0.0000	0.0000	0.0000	0.0000	0.0000	0.0000
	Tubers	0.0035-0.0119	0.0021-0.0047	0.0012-0.0027	0.0006-0.0018	0.0011-0.0026	0.0009-0.0043
	Vegetables	0.0000-0.0055	0.0000-0.0031	0.0000-0.0028	0.0000-0.0023	0.0000-0.0031	0.0000-0.0032
Livestock products	Eggs	0.0012-0.0104	0.0010-0.0086	0.0006-0.0052	0.0003-0.0028	0.0003-0.0027	0.0002-0.0016
	Meats	0.0001-0.0013	0.0002-0.0012	0.0002-0.0016	0.0001-0.0015	0.0001-0.0008	0.0000-0.0002
Fishery products	Crustaceans	0.0000-0.0000	0.0000-0.0000	0.0000-0.0000	0.0000-0.0000	0.0000-0.0000	0.0000-0.0000
	Fishes	0.0000-0.0002	0.0000-0.0001	0.0000-0.0001	0.0000-0.0000	0.0000-0.0001	0.0000-0.0001
	Cephalopoda	0.0000-0.0001	0.0000-0.0001	0.0000-0.0001	0.0000-0.0001	0.0000-0.0001	0.0000-0.0001
	Shellfishes	0.0000-0.0004	0.0000-0.0003	0.0000-0.0001	0.0000-0.0001	0.0000-0.0001	0.0000-0.0001
	Sea algae	0.0000-0.0006	0.0000-0.0004	0.0000-0.0002	0.0000-0.0001	0.0000-0.0002	0.0000-0.0002
Processed products	Beverages	0.0000-0.0017	0.0000-0.0016	0.0000-0.0007	0.0000-0.0006	0.0000-0.0003	0.0000-0.0001
	Other processes products	0.0000-0.0003	0.0000-0.0005	0.0000-0.0009	0.0000-0.0005	0.0000-0.0014	0.0000-0.0016

Table S9. Instrumental conditions of ICP-MS for the determination of Tl.

Parameters	Conditions
Instrument	Agilent ICP-MS 7700x
R.F. Matching	27.12 MHz
R.F. Power	1550 W
Carrier gas	Ar 1.10 L min $^{-1}$
Collision mode	He 4.0 mL/min $^{-1}$
Ion monitored	m/z 203, 205
Lens voltage	10 V
Plasma gas	15 L min $^{-1}$
Axillary gas	1.0 L min $^{-1}$

Figure S1. Fish-bone diagram for Tl analysis uncertainty for rice.

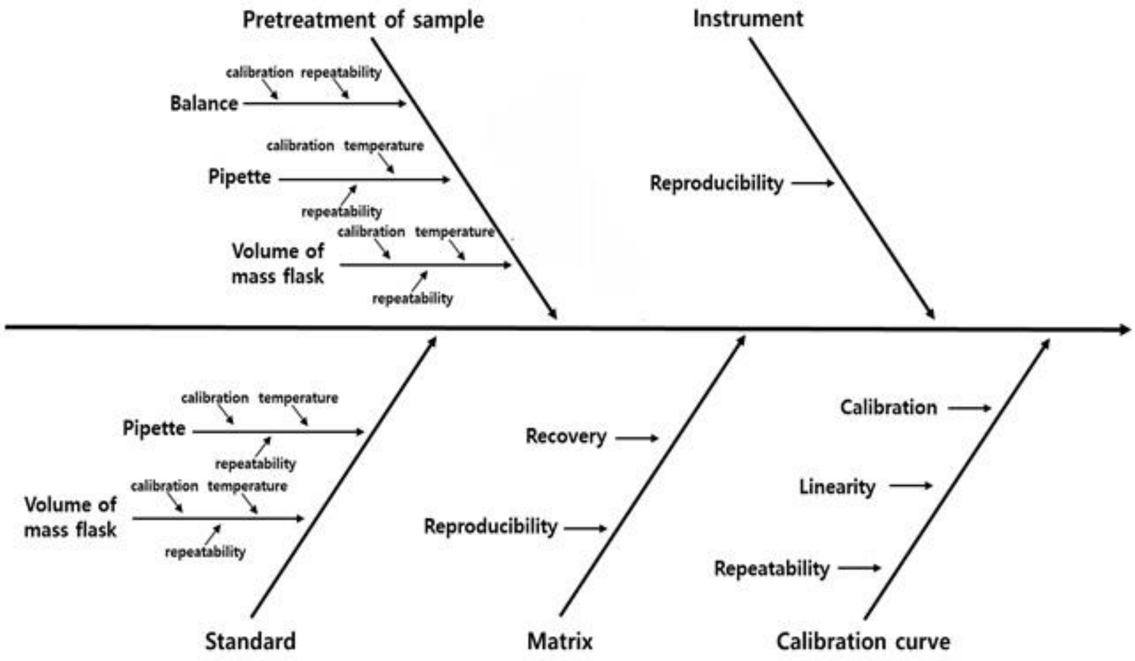

Supplement: Supplementary file 1 [file molecules-26-06729-s001.zip › molecules-1422067-supplementary.pdf]
